# The TRUST trial in ovarian cancer: a missed opportunity or a turning point? The position of the Italian MITO group

**DOI:** 10.3389/fonc.2026.1853078

**Published:** 2026-05-28

**Authors:** Martina Arcieri, Sandro Pignata, Domenica Lorusso, Anna Fagotti, Vito Chiantera, Antonino Ditto, Giorgio Valabrega, Claudia Marchetti, Claudio Zamagni, Valentina Tuninetti, Alberto Farolfi, Stefano Restaino, Giuseppe Vizzielli

**Affiliations:** 1 Department of Obstetrics and Gynaecology, Azienda Sanitaria Universitaria Friuli Centrale, Ospedale Santa Maria della Misericordia, Udine, Italy; 2 Department of Urology and Gynecology, Istituto Nazionale Tumori IRCCS Fondazione G. Pascale, Naples, Italy; 3 Department of Biomedical Sciences, Humanitas University, Milan, Italy; 4 Department of Gynecologic Oncology, Humanitas San Pio X, Milan, Italy; 5 Gynecologic Oncology Unit, Department of Women, Children and Public Health Sciences, Fondazione Policlinico Universitario Agostino Gemelli IRCCS, Rome, Italy; 6 Catholic University of the Sacred Heart, Rome, Italy; 7 Gynecologic Oncology Unit, Centro di Riferimento Oncologico, National Cancer Institute, Aviano, Italy; 8 Department of Oncology, University of Turin, Medical Oncology, Ordine Mauriziano Hospital, Turin, Italy; 9 Division of Oncology, IRCCS Azienda Ospedaliero-Universitaria di Bologna, Bologna, Italy; 10 Medical Oncology, Breast & GYN Unit, IRCCS Istituto Romagnolo per lo Studio dei Tumori (IRST) “Dino Amadori”, Meldola, Italy; 11 PhD School in Biomedical Sciences, Gender Medicine, Child and Women Health, University of Sassari, Sassari, Italy; 12 Department of Medicine, University of Udine, Udine, Italy

**Keywords:** Cytoreduction, neoadjuvant chemotherapy, ovarian cancer, randomized clinical trial, surgery

## 1 Introduction

**Figure 1:**
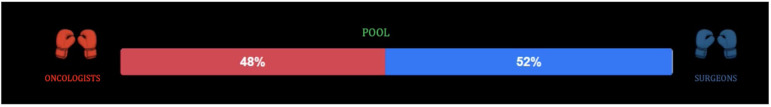
Results of MITO debate.

On 24 June 2025, the MITO (Multicenter Italian Trials in Ovarian Cancer and Gynecologic Malignancies) group hosted a webinar entitled “*TRUST on the Ring*” to discuss the TRUST trial (1, 2), presented at ASCO (American Society of Clinical Oncology) 2025 conference. Eight leading Italian experts (four surgeons and four medical oncologists) engaged in a structured debate attended by more than 150 participants. A final poll split 52% vs 48% between surgical and oncologic perspectives, highlighting ongoing equipoise but shared agreement on the central role of surgery in ovarian cancer management (**Figure 1**).

The TRUST trial compared primary debulking surgery with neoadjuvant chemotherapy followed by interval debulking surgery in FIGO (The International Federation of Gynecology and Obstetrics) stage IIIB–IV ovarian cancer.

Conducted in high-volume expert centers, it did not meet its primary endpoint of overall survival (54.3 vs 48.3 months; hazard ratio (HR) 0.89; p=0.24). However, progression-free survival significantly favored primary debulking surgery (22.2 vs 19.7 months; HR 0.80; p=0.02), with <1% perioperative mortality, higher morbidity, but no quality-of-life difference.

Although labeled a “negative study”, the trial renewed debate on optimal treatment in advanced ovarian cancer.

## 2 Discussion

The MITO discussion, pending for the complete peer-reviewed publication of trial, highlighted three fundamental limitations:

### 2.1 Inadequate surgical candidate selection

TRUST did not require the use of objective selection tools for primary debulking surgery eligibility. Validated laparoscopic scoring systems like the Fagotti Predictive Index Value (3) and the Vizzielli score (4) can reliably predict resectability and estimate the risk of postoperative complications. For over a decade in Italy, these validated tools have guided selection of patients most likely to achieve complete resection with acceptable morbidity. Their absence in TRUST may have reduced primary debulking surgery effectiveness, underscoring the need for reproducible instruments rather than reliance on individual surgeon experience.

### 2.2 Limitations of overall survival as the sole primary endpoint

The overall survival remains the gold standard endpoint for prospective randomized studies; however, it is particularly challenging to demonstrate its benefits in frontline trials involving populations with prolonged post-progression survival, especially in the current context where both medical and surgical strategies after first progression have been shown to influence overall survival. Unequal access to Poly ADP-ribose polymerase inhibitors, differences in secondary cytoreduction use, and the confounding effects of sequential therapies likely obscure potential overall survival benefits. In TRUST, median overall survival in the primary debulking surgery arm was less favorable than in maintenance trials like SOLO-1 (5) and PRIMA (6), reflecting the evolving treatment landscape. Progression-free survival —arguably more directly influenced by the initial treatment strategy— favored primary debulking surgery in TRUST. The inclusion of both overall survival and progression-free survival as co-primary endpoints could have provided a more comprehensive assessment.

### 2.3 Underpowered and non-stratified subgroup analyses

Data interpretation is limited by the high proportion and heterogeneity of FIGO stage IV patients, as well as the lack of BRCA/HRD stratification. This represents a significant limitation, as these molecular features are well-established determinants of prognosis and treatment response, particularly in the context of maintenance therapies.

## 3 Clinical interpretation and risk of misapplication

The debate reinforces that neither primary debulking surgery nor neoadjuvant chemotherapy should be considered universally superior. Two key considerations emerged from the MITO discussion: (1) in expert hands and with proper selection based on validated and reproducible instruments such as Fagotti Predictive Index Value, primary debulking surgery remains effective and safe, as shown by the progression-free survival benefit; (2) excessively aggressive surgery with high morbidity is unjustifiable as complications delay effective therapy and worsen prognosis. A consensus is required to identify procedures that are oncologically meaningful versus those carrying excessive risk.

The progression-free survival advantage observed suggests that primary debulking surgery may still offer a clinically meaningful benefit when reserved for carefully selected patients. The optimal candidate for upfront surgery likely includes patients with good performance status (ECOG <2), disease not extending to FIGO stage IV, limited need for extensive visceral resections, and a high likelihood of achieving complete cytoreduction, as predicted by validated laparoscopic scoring systems and expert preoperative assessment. Conversely, attempting highly aggressive surgery in frail patients or in cases with a low probability of complete resection may expose patients to unjustifiable morbidity, delayed systemic therapy, and potentially worse oncologic outcomes.

These conclusions highlight the need the importance of centralizing care in high-volume centers to ensure appropriate patient–treatment matching.

A potential misinterpretation, especially in low-volume settings, could be to interpret TRUST as a justification for the universal use of neoadjuvant chemotherapy/interval debulking surgery, regardless of whether the tumor can be fully removed initially. This could diminish the importance of high-quality primary debulking surgery when it is still feasible and advantageous. Additionally, in centers lacking the right surgical expertise and specialized knowledge, interval debulking surgery might be mistakenly seen as a simpler surgery compared to primary debulking surgery and could be improperly used as a reason to move treatments that require dedicated expertise and care knowledge.

In Italy, it is unlikely that TRUST will change practice in referral centers where structured selection with laparoscopic scoring and multidisciplinary assessment ensures appropriate triage, offering a model that could guide international practice.

Beyond national boundaries, TRUST serves as a reminder that trial design must evolve alongside treatment advances.

## 4 Contextualization within existing evidence

The findings of TRUST should be interpreted alongside previous randomized trials, such as EORTC 55971 (7), CHORUS (8), SCORPION (9) and JCOG0602 (10), which demonstrated non-inferiority of neoadjuvant chemotherapy compared with primary debulking surgery, particularly in less selected populations. Importantly, surgical outcomes remain highly dependent on institutional expertise, limiting the generalizability of results across different healthcare settings. Furthermore, emerging data from our recent meta-analysis (11) of trials comparing primary debulking surgery and interval debulking surgery indicate that no clinically meaningful OS advantage for upfront surgery has been consistently demonstrated. However, these trials are affected by substantial methodological heterogeneity and limitations. Future studies must be adequately powered, methodologically rigorous, and conducted within certified surgical networks to determine whether specific patient subgroups derive differential benefit from treatment sequencing.

## 5 Future directions and conclusions

The TRUST trial should not be interpreted as definitively supporting one treatment paradigm over another. Rather, it underscores the need for a more individualized approach to advanced ovarian cancer.

Future studies should incorporate:

- objective selection criteria (laparoscopic scoring, imaging-based predictive models);
- stratification by patients’ performance status, molecular markers (BRCA, HRD) and disease stage;
- endpoints sensitive to early treatment effects (PFS, time to second progression), complemented by OS and patient-reported outcomes;
- detailed reporting of surgical complexity and morbidity.

Only through standardized and biologically informed trial designs will it be possible to move beyond apparent therapeutic equivalence toward a truly personalized treatment strategy.

The “TRUST on the Ring” debate highlights both the complexity of the evidence and the persistence of clinical uncertainty.

Moving forward, the key challenge is to build consensus pathways that deliver personalized, evidence-based care while balancing benefit, safety, quality of life, and health system realities.
